# Progesterone Suppresses *Neisseria gonorrhoeae*-Induced Inflammation Through Inhibition of NLRP3 Inflammasome Pathway in THP-1 Cells and Murine Models

**DOI:** 10.3389/fmicb.2021.570093

**Published:** 2021-02-09

**Authors:** Song Zhang, Yingmiao Zhang, Lu Gan, Fen Wei, Bao Chai, Amaneh Abdel Hafez A Aljaafreh, Xinxin Liu, Xiaoru Duan, Jian Jiang, Xin Wang, Mengwen He, Xian Huang, Huahua Cai, Tie Chen, Hongxiang Chen

**Affiliations:** ^1^Department of Dermatology, Union Hospital, Tongji Medical College, Huazhong University of Science and Technology, Wuhan, China; ^2^Department of Clinical Immunology, Tongji Hospital, Tongji Medical College, Huazhong University of Sciences and Technology, Wuhan, China; ^3^Department of Clinical Laboratory, The Central Hospital of Wuhan, Tongji Medical College, Huazhong University of Science and Tchnology, Wuhan, China; ^4^Department of Dermatology, Zhongnan Hospital, Wuhan University, Wuhan, China; ^5^Department of Dermatology, The 6th Affiliated Hospital of Shenzhen University Health Science Center, Shenzhen, China; ^6^Department of Dermatology, Huazhong University of Science and Technology Union Shenzhen Hospital, Shenzhen, China; ^7^Department of Dermatology, Affiliated Hospital of Nantong University, Nantong, China

**Keywords:** *Neisseria gonorrhoeae*, inflammation, NLRP3 inflammasome, NF-κB, reactive oxygen species

## Abstract

Asymptomatic/subclinical gonococcal infections in females continue to be prevalent within the general population, thus emerging as a global health problem. However, the reasons for these clinical manifestations are unknown. Our group had previously found out that in females, asymptomatic gonococcal infections correlate with higher serum progesterone (P4) levels and lower IL-1β levels in cervical secretions. We used murine infection model and THP-1 cells to determine whether P4 exerts anti-inflammatory effects on gonococcal infections. In the murine infection model, P4 (1 mg/day) inhibited the inflammatory effects induced by gonococcal infections which led to decreased neutrophil infiltration, reduced polymorphonuclear neutrophils (PMNs) numbers, IL-1β, TNF-α, and IL-6 levels in vaginal secretions. In addition, P4 down-regulated the mRNA and protein levels of NLRP3, associated with lower mRNA levels of pro-IL-1β, repressed caspase-1 activity in genital tissues and THP-1 cells. Moreover, P4 suppressed the phosphorylation levels of NF-κB and attenuated *Neisseria gonorrhoeae* (*N. gonorrhoeae*, gonococci or GC)-induced ROS generation. This is consistent with the two signals required for activation of the NLRP3 (NOD-, LRR-, and pyrin domain-containing protein 3) inflammasome. In conclusion, our result shows that P4 suppresses the gonococci induced-inflammation, especially through the NLRP3 inflammasome pathway, and partially explains the pathogenesis of asymptomatic GC infection in women.

## Introduction

It has long been acknowledged that the susceptibility and immune response to *Neisseria gonorrhoeae* (*N. gonorrhoeae*, gonococci, or GC) infection between men and women are different(Kent et al., 2005; McMurray et al., 2018). Asymptomatic/subclinical gonococcal infections occur more frequently in female (50–80%) (Pedersen and Bonin, 1971; McCormack et al., 1977) compared to male (1–3%) (Handsfield et al., 1974; Upchurch et al., 1990); thus, the exact incidence of gonococcal infections is severely underestimated. If left untreated, asymptomatic/subclinical infections with GC may cause continued prevalence and severe complications, such as pelvic inflammatory disease (PID), infertility, and ectopic pregnancy (Unemo et al., 2017).

The reasons for these different clinical manifestations are manifold and mostly not clear. In addition to the gender-specific physiological structures that mediate sex-specific responses to pathogens, gender-related changes in sex steroid levels may also play an important role (van Lunzen and Altfeld, 2014). Cervical secretions are more often cultured positive of gonococci when women are in the proliferative stage of their menstrual cycle (James and Swanson, 1978; Overton et al., 2008). Female mice are more susceptible to sexually transmitted pathogens during the diestrus phase of the murine estrous cycle when progesterone (P4) levels are the highest (Kaushic et al., 2003; Parr and Parr, 2003). P4 could increase gonococcal survival within epithelial cells, thereby inhibiting the release of bacteria (Edwards, 2010) and consequently downregulating the bacteria loads in mouse vagina (Cole et al., 2010). Our group has also discovered that the average serum P4 levels in the asymptomatic female gonorrhea patients were significantly higher than that in the symptomatic group (Wu et al., 2011).

One of the reason of this phenomenon might be that P4 is one of the immune-suppressive hormones which regulates the innate and adaptive immune function of female reproductive tract (Zhang et al., 2006). In innate immunity, P4 downregulates IFN-γ secretion in human peripheral blood natural killer cells (Robinson and Klein, 2012) and weaken macrophage functions (Franchi et al., 2012). In adaptive immunity, P4 suppresses the CD4^+^ T cells proliferation, inhibits the cytotoxic activity of CD3^+^ CD8^+^T cells (García et al., 2016), and interferes with the polarization of Th1/Th17 cells (Andrabi et al., 2017). Therefore, the immune suppression ability of P4 suggests that it may participate in the pathogenesis of female asymptomatic gonococcal infections.

It is known that gonococci can repeatedly infect individuals without any development of immunological memory (Quillin and Seifert, 2018). *N. gonorrhoeae* mainly induces the congenital immune signaling pathways (Pekmezovic et al., 2019). LOS (lipo-oligosaccharides) is an important virulence factor of *N. gonorrhoeae*, which includes lipid A and oligosaccharide structure (Pridmore et al., 2003). It has been reported that the lipid A molecule activates toll-like receptor 4 (TLR4), and the oligosaccharide structure activates C-type lectin receptors (van Vliet et al., 2009). Recent study also demonstrated that *N. gonorrhoeae* activates the NLRP3-inflammasome through undetermined mechanisms (Duncan et al., 2009). The inflammasome is a cytosolic protein complex which regulates innate immunity and inflammation (Martinon et al., 2009). There are two signals to fully activating the NLRP3 inflammasome: activation of nuclear factor-kappa B (NF-κB), inducing synthesis of pro-IL-1β and NLRP3; danger-associated molecular patterns (DAMP), such as ROS generation that lead to activation of pro-caspase-1 to caspase-1, and cleavage of pro-IL-1β to mature IL-1β (Swanson et al., 2019).

Recent studies have revealed that P4 inhibits NF-κB activation in myometrial cells and TLR4-mediated TNF-α production in macrophages (Jones et al., 2008; Tamura et al., 2011). P4 can attenuate the release of mitochondrial ROS and blocks the mitochondrial permeability transition pore (mPTP) in mouse models (García et al., 2016). Although there has been evidence showing that *N. gonorrhoeae* could activate NLRP3 inflammasome (Li et al., 2019), the relationship between inflammasome induced by gonococci and P4 remains unknown.

We had previously found that cervical secretion of IL-1β in asymptomatic gonorrhea patients were much less compared to that in symptomatic patients (Wu et al., 2011). Therefore, we speculated that the P4 plays an anti-inflammatory role via inhibition of inflammasome activation. In the present study, we demonstrated that the P4 inhibits *N. gonorrhoeae*-induced NLRP3 activation and IL-1β maturation in THP-1 cells and in murine models. Further experiments showed that the P4 inhibits the NF-κB signal pathway and downregulate ROS production, consistent with the two signals required by activation of the NLRP3 inflammasome.

## Materials and Methods

### Mice and Reagents

BALB/c mice used in this experiment were purchased from Beijing HFK Bioscience Co., Ltd. (Beijing, China). They were then bred in the animal facility under specific pathogen-free (SPF) conditions for more than 1 week before experiment. Mice were age- and weight-matched when used in experiments. Animal experiments was performed in the USUHS laboratory animal facility. The protocol used in these experiments had been approved by the USUHS Institutional Animal Care and Use Committee.

Chemicals including 17β-estradiol, progesterone, Phorbol 12-myristate 13-acetate, hydrogen peroxide, DCFH-DA, Hoechst 33342, LPS and 2-mercaptoethanol were obtained from Sigma Inc., United States. GC agar, GC-VCNTS agar, and iso-vitalex were from Difco, BD, United States. Anti-caspase-1p20, anti-caspase-1, anti-GAPDH mAb were obtained from Santa Cruz, CA, United States. Anti-NLRP3 mAb, DyLight 594 conjugated goat anti-mouse IgG and DyLight 488 conjugated goat anti-rabbit IgG were purchased from Abcam, MA, United States. Anti-phospho-NF-κB and anti-NF-κB mAb were from Cell Signaling Technology, Danvers, MA, United States. PCR primers were purchased from Takara Biotechnology, Dalian, China.

### Bacterial Strains and Culture Conditions

Gonococcal strain MS11 with piliated-negative colony morphology and lacto-N-neotetraose (LOS b) phenotypes were used in all experiments. Strain MS11 were provided by professor Tie Chen (Zhang et al., 2005). The strain was cultured on GC agar with iso-vitalex at 37°C in 5% CO_2_ and maintained as previously described (Zhang et al., 2005). *N. gonorrhoeae* was isolated from vaginal mucus using GC-VCNTS agar.

### Murine Infection Models

The 17β-estradiol was used to treat female intact and ovariectomized (Ovx-)BALB/c mice (4–6 weeks old) in the diestrus of the estrous cycle to increase long susceptibility period to *N. gonorrhoeae*. Fourteen days before the experiment, we surgically removed the ovaries of the Ovx- mice. The infection process was similar to the description given in Jerse’s paper (Jerse et al., 2011). The mice were intravaginally inoculated with 100 μl of phosphate buffer saline (PBS) (control) or saline containing 10^6^ CFUs of *N. gonorrhoeae*. Mice were subcutaneously injected with 17β-estradiol 2 days before inoculation (day -2), on the day of bacterial inoculation (day 0) and 2 days after inoculation (day +2). Post inoculation, progesterone (1 mg/day) was injected intramuscularly for 11–17 consecutive days until the vaginal smears were cultured negative. BALB/c mice were randomly divided into the following groups with five mice per group: intact group (intact mice which were intravaginally inoculated with GC); Ovx-group (Ovx-mice which were intravaginally inoculated with GC); Ovx- + P4 group [Ovx-mice which were intravaginally inoculated with GC, with P4 (1 mg/day) injected intramuscularly]; control group (intact mice which were intravaginally inoculated with PBS). To prevent commensal flora overgrowth, antibiotics were injected intraperitoneally as described (Jerse et al., 2011). Then we continuously sampled the vaginal mucus from test and control mice for 10–12 days. A portion of vaginal mucus was quantified on GC-VCNTS agar. Another part of the mucus was stained and calculated the number of PMNs in 100 vaginal cells. Five days post inoculation, several mice were sacrificed for further study.

### Histological Analysis

Surgical specimens of the vaginal and cervical tissues were paraffin-embedded for H&E staining. Paraffin tissues specimens (4 μm) were stained with hematoxylin (Beyotime, Shanghai, China) for 40 s and with eosin (Beyotime, Shanghai, China) for 30 s. The tissue sections were examined under an OLYMPUS light microscope.

Mice’s vaginal and cervical tissue were paraffin-embedded, cut, air-dried, fixed with acetone, and stained with anti-caspase-1p20, anti-NLRP3, anti-CD68, anti-IL-1 β mAb. Then slides were incubated with DyLight 594 conjugated goat anti-mouse IgG and DyLight 488 conjugated goat anti-rabbit IgG for 1 h at room temperature. Next, the slides were washed three times and incubated with Hoechst 33342. Fluorescence images were observed by the OLYMPUS fluorescence microscope. Fluorescence images were quantified using ImageJ software (from NIH and available at https://imagej.nih.gov/ij/) to evaluate the relative fluorescence intensity.

### Cytokine Detection by Multi-Analyte Flow Assay Kit

Fifty microliter of vaginal mucus was continuously collected from test and control mice for 10–12 days. To detect the IL-1β, TNF-α, and IL-6 levels in vaginal mucus, commercially available LEGEND plex^TM^ Multi-Analyte Flow Assay Kits (Biolegend, San Diego, CA, United States) in accordance to the manufacturer’s instructions were used (Araújo et al., 2015).

### Cell Lines and Cell Stimulation

Human THP-1 cells were provided by Cell Bank of Academy of Sciences (Shanghai, China), grown in an RPMI 1,640 medium and supplemented with 10% FBS and 50 μM 2-mercaptoethanol. All cultures were incubated at 37°C under 5% CO_2_/95% air. THP-1 cell line used in this study were regularly tested for mycoplasma.

For the induction of cell differentiation, THP-1 cells were stimulated for 72 h with 200 nM PMA. After resting another 24 h, 1 × 10^6^ cells were pre-treated with *N. gonorrhoeae* for 4 h. Subsequently, the cells were stimulated with P4. As a control, H_2_O_2_ was added at the same concentration in the medium of THP-1.

### Reverse Transcription and Real-time PCR Analysis

RT-PCR was performed according to the manufacturer’s instructions. Briefly, TRIzol (Invitrogen, Melbourne, VIC, Australia) were used to isolate the total RNA. Revert Aid First Strand cDNA Synthesis Kit (K1622, Thermo Fisher Scientific, United States) were used to synthesize the cDNA. Quantitative real-time PCR was performed using the SYBR Green kit (Takara Biotechnology, Kyoto, Japan) on a real-time PCR system (StepOnePlus^TM^ Real-Time PCR System, Thermo Scientific). The reaction was performed with a denaturation step at 95°C for 30 s, annealing at 60°C for 30 s, and extension at 72°C for 30 s for 45 cycles. Relative amounts of target mRNA were normalized to GAPDH mRNA levels (the internal control). Primers used in this experiment showed below: NLRP3 human, F: 5′-CCCCGTGAGTCCCATTA-3′; NLRP3 human, R: 3′-GACGCCCAGTCCAACAT-5′; NLRP3 mice, F:5′-TCCACAATTCTGACCCACAA-3′; NLRP3 mice, R:3′-ACCTCACAGAGGGTCACCAC-5′; pro-IL-1β human, F:5′ -CCTGTGGCCTTGGGCCTCAA-3′; pro-IL-1β human, R:3′ -GGTGCTGATGTACAGTTGGG-5′; pro-IL-1β mice, F:5′-TC TTTGAAGTTGACGGACCC-3′; pro-IL-1β mice, R:3′-TGAG TGATACTGCCTGCCTG-5′. RelA/p65 human, F:5′-CGAATGG CTCGTCTGTAGTGCA-3′, R: 5′-TGCGCTGACTGATAGCC TGCTCCAGGT-3′; GAPDH mice, F:5′-AGAGGGAAATCG TGCGTGAC-3′; GAPDH mice, R:3′-CAATAGTGATGACCT GGCCGT-5′; GAPDH human, F: 5′-GTCTCCTCTGACTTC AACAGCG-3′; GAPDH human, 3′-ACCACCCTGTTGCTGT AGCCAA-5.

### Cytokine Detection by Enzyme-Linked Immunosorbent Assay (ELISA)

Cytokines IL-1β, IL-6, TNF-α from cell culture supernatants were assayed by ELISA kits following the manufacturers’ instructions (MultiSciences, Hangzhou, China).

### Western Blot

Adherent cells were harvested and lysed in lysis buffer (50 mM Tris-HCl, 150 mM NaCl, 1% Non-idet P-40, 0.1% SDS, 0.5% sodium deoxycholate, and 1 mM phenylmethysulfonyl fluoride). For detection of the protein concentration, a Bio-Rad protein assay kit was used, with bovine serum albumin acting as a reference. 10% SDS-PAGE gel (Bio-Rad) were used to separate the protein sample containing 10 μg of protein, and then it was electrophoretically transferred onto the PVDF membrane (0.45 μm thick). After blocking with 5% non-fat milk, membranes were incubated with primary antibodies including anti-phospho-NF-κB, anti-NF-κB, anti-NLRP3, anti-caspase-1, anti-IL-1β, anti-GAPDH mAb, followed by incubation with a horseradish peroxidase-conjugated secondary antibody and visualized using a Bio-Rad Chemi Doc XRS Imaging System with an XRS camera (Bio-Rad, Hercules, CA, United States).

### Measurement of Intracellular Reactive Oxygen Species (ROS)

Intracellular ROS levels were measured using oxidation sensitive fluorescent dyes, DCFH-DA. Cells were incubated with 10 μM DCFH-DA (Beyotime, Shanghai, China) for 20 min at 37°C and subsequently washed. The cells were then stimulated with 1 μg/ml of ultrapure LPS for 1 h, and ROS generation was measured from dichlorofluorescein (DCF) fluorescence intensity by OLYMPUS fluorescence microscope.

### Statistical Analyses

Western blot and immunofluorescent experiments were performed three times and data represent the mean values ± *SD*. Statistical comparisons between two groups were performed using a Student’s *t*-test. GraphPad Software Prism 6.0 was used for statistical analysis. *P* < 0.05 were considered significant.

## Results

### P4 Suppresses *N. gonorrhoeae*-Induced Inflammation in Genital Tissues

**FIGURE 1:**
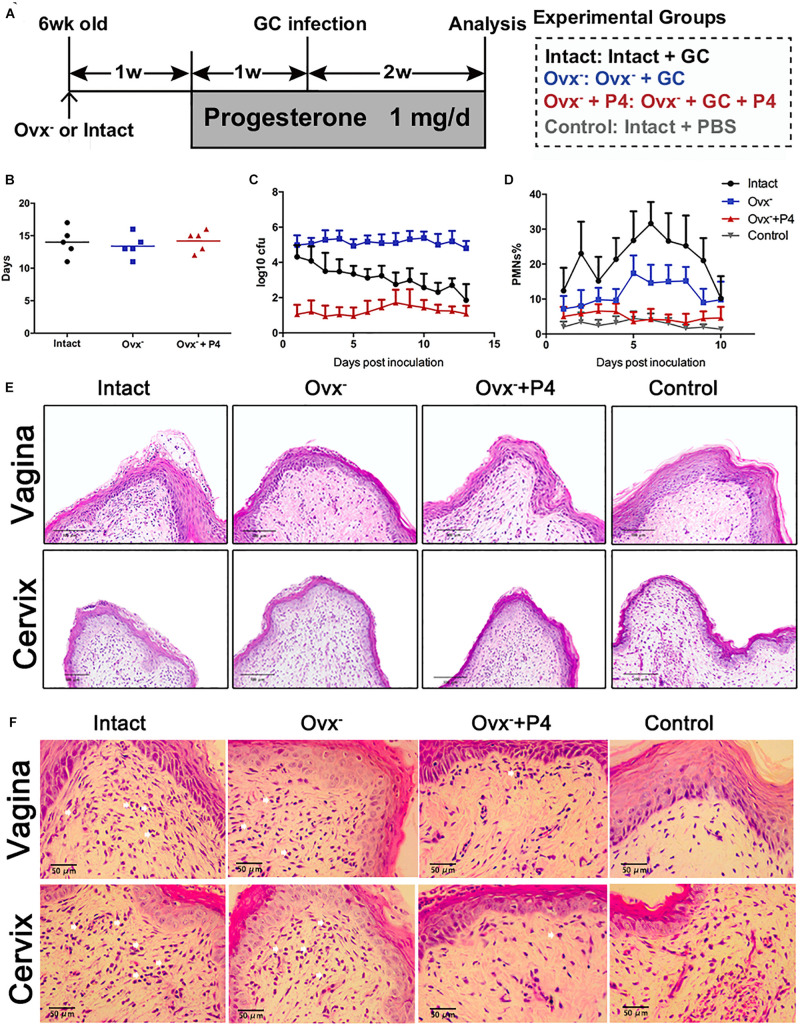
Progesterone suppresses *N. gonorrhoeae*-induced neutrophils infiltration in murine models. **(A)** Time line for the *N. gonorrhoeae* infection model. **(B)** The average duration of gonococci recovery among the three groups. **(C)** The average number of gonococci recovered from vaginal swabs of BALB/c mice, the single vaginal swab was suspended in 100 μl of PBS. **(D)** The percent of PMNs in stained vaginal smears from experimental mice were obtained on fifth day after inoculation. **(E,F)** Paraffin embedded sections (vaginal and cervical tissues of infected mice and uninfected controls) were stained with hematoxylin and eosin, and tissue sections were examined under an OLYMPUS light microscope. All data are representative of at least three independent experiments. Data are presented as the mean ±*SD*, *n* = 5. OVX^–^, ovariectomy; GC, *Neisseria gonorrhoeae*; P4, progesterone; CFU, Colony-Forming Units.

To determine whether or not the P4 suppresses the inflammation induced by gonococci *in vivo*, we established 17β-estradiol treated female BALB/c mice with vaginal infection of *N. gonorrhoeae*. Schematic of mouse infection protocol has been shown in Figure 1A. The experiments were carried out in four groups. High levels of bacteria in the vaginal secretions were detected on the 5th day after inoculation in each group, suggesting that the *N. gonorrhoeae*-infected mice had been successfully established (Figures 1B,C). No significant differences were found on the average duration of gonococci recovery among the three groups (*p* 0.6971 and *p* 0.5653, respectively) (Figure 1B). For the control group, the average duration of gonococci recovery was 14.0 days (range, 13–15 days). For the Ovx^–^ group, the average duration was 13.4 days (range, 12.6–14.2 days). For the Ovx^–^ + P4 group, the duration was 14.2 days (range, 13.5–14.9 days) (Figure 1B). Data showed that, among these 4 groups, the average number of gonococci recovered from vaginal swabs of BALB/c mice in the Ovx^–^ + P4 group was lower than the other groups, indicating that P4 inhibit the growth of GC in mouse vagina (Figure 1C).

To investigate the local *N. gonorrhoeae* induced-inflammation, polymorphonuclear neutrophils (PMNs) numbers were estimated in genital tract tissues and smears obtained from vaginal swab. As shown in Figures 1D,E, PMNs were increased most significantly in the intact group out of the four groups. The PMNs secreted by Ovx^–^ + P4 group were lower than the intact group and the Ovx^–^ group, which was basically equivalent to the control group. To further investigate the number of PMNs in mouse models, vaginal and cervical tissues were obtained on the 5th day after inoculation. In mucosal and submucosal layers of vagina and cervix, increased levels of PMNs were present in inoculated (day 5) mice. Meanwhile, levels of PMNs in P4 treated mice were lower compared to inoculated mice (Figure 1E).

**FIGURE 2:**
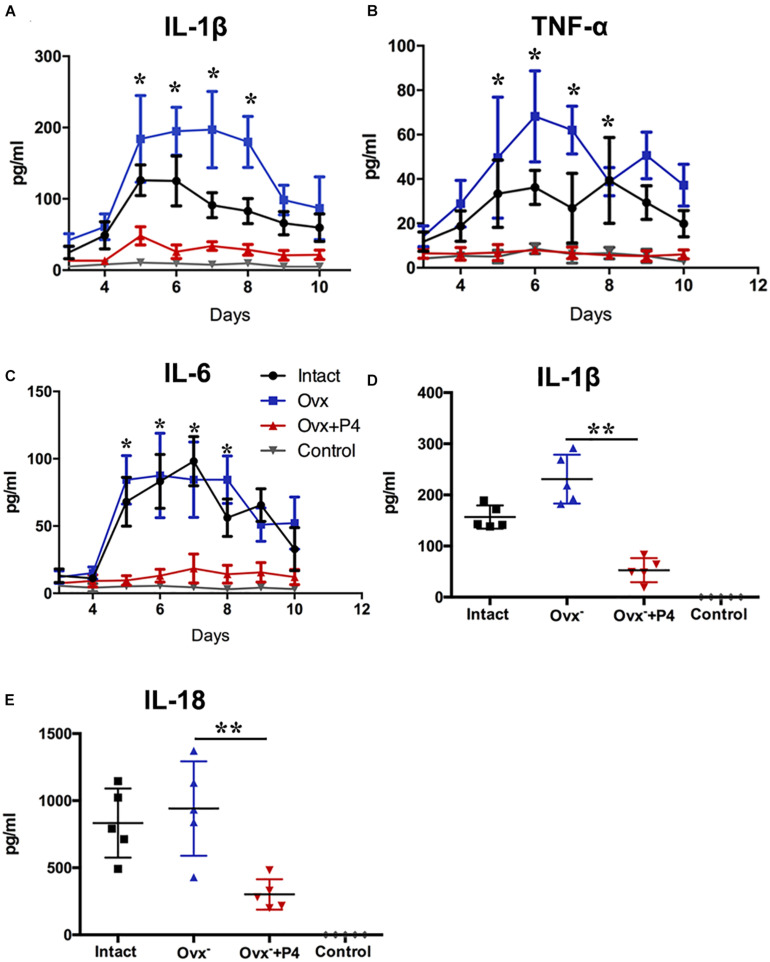
Progesterone suppresses *N. gonorrhoeae*-induced cytokines secretion in murine models. 50 μl of vaginal mucus was continuously collected from test and control mice for 10–12 days, and detection of IL-1β **(A)**, TNF-α **(B)**, and IL-6 **(C)** levels were carried out using commercially available LEGEND plex^TM^ Multi-Analyte Flow Assay Kits. The IL-1β **(D)** and IL-18 **(E)** levels from the vaginal secretions of *N. gonorrhoeae* infected mice on fifth day after inoculation. All data are representative of at least three independent experiments. *p < 0.05, **p < 0.01. Data are presented as the mean ± *SD*, *n* = 5. Ovx^–^, ovariectomy; P4, progesterone.

To detect the level of pro-inflammatory cytokines in mice vagina, we collected their vaginal secretions from infected mice every day for 10–12 consecutive days. The result showed that IL-1β, IL-6, and TNF-α levels peaked in the infected groups on the fifth day (Figures 2A–C). The IL-1β (Figure 2A), IL-6 (Figure 2B), and TNF-α (Figure 2C) levels in the vaginal secretions in intact group were significantly higher than that observed in control group. The Ovx^–^ group was slightly lower than that in the intact group. Our data also showed that the levels of inflammatory cytokines were obviously decreased in Ovx^–^ + P4 group. These results suggest that P4 down regulates the PMNs and inflammatory cytokines secretion in genital tract caused by gonococci in murine models.

### P4 Suppresses *N. gonorrhoeae*-Induced NLRP3 Inflammasome Activation in Genital Tissues

**FIGURE 3:**
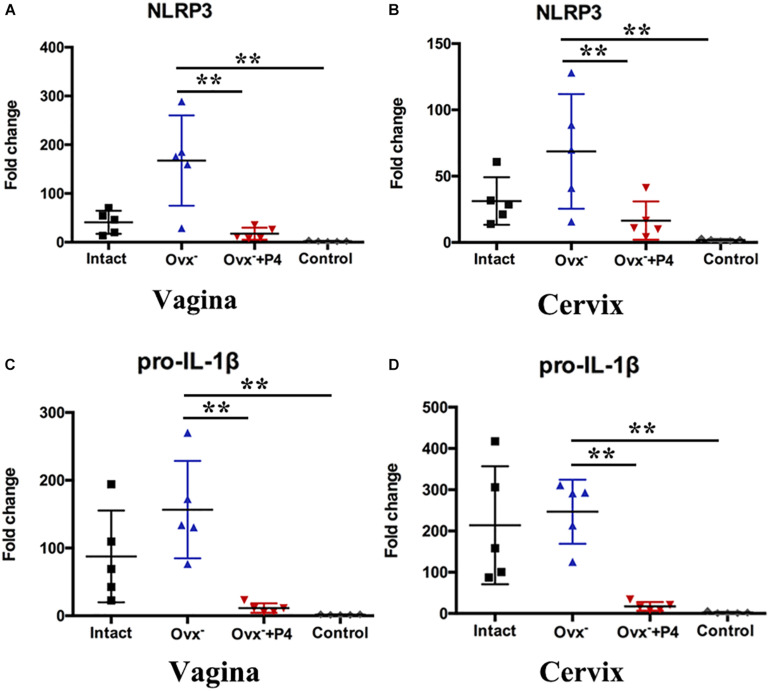
Progesterone suppresses *N. gonorrhoeae*-induced NLRP3 inflammasome activation in murine models. The mRNA levels of NLRP3 were assayed in the vaginal **(A)** and cervical **(B)** tissues on day 5 after inoculation. The mRNA levels of pro-IL-1β were assayed in the vaginal **(C)** and cervical **(D)** tissues on day 5 after inoculation. All data are representative of at least three independent experiments. **p < 0.01. Data are presented as the mean ± SD, *n* = 5. Ovx^–^, ovariectomy; P4, progesterone.

**FIGURE 4:**
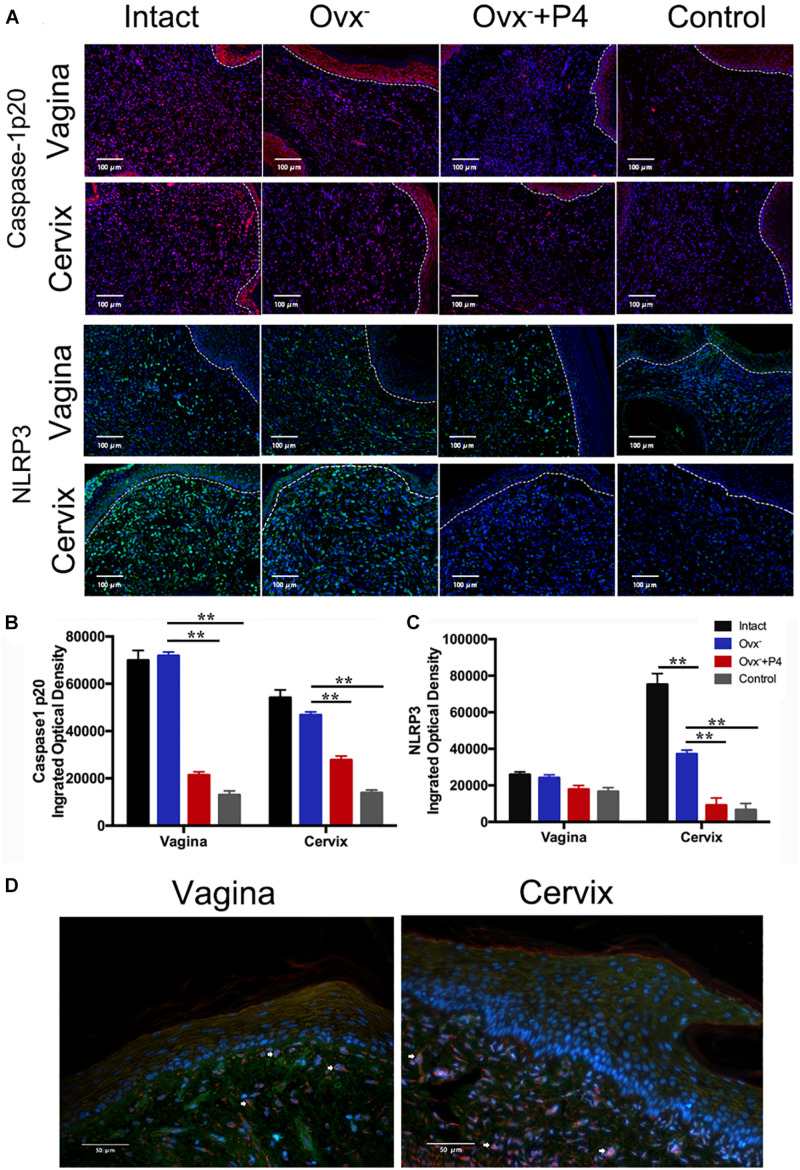
Immunofluorescence of NLRP3 and Caspase-1 p20 in vaginal and cervical tissues. **(A)** Immunofluorescence and fluorescence microscope were used to estimate the number of NLRP3-positive cells (red fluorescence) and caspase-1p20-positive cells (green fluorescence) on day5 after inoculation. **(B)** Semi-quantification analysis of relative fluorescence intensity of NLRP3 using ImageJ software. **(C)** Semi-quantification analysis of relative fluorescence intensity of caspase-1p20 using ImageJ software. **(D)** Immunofluorescence of anti-CD68 (red fluorescence) and anti-IL-1β (green fluorescence) of vaginal and cervical tissues. **p < 0.01. Data are presented as the mean ± *SD*, *n* = 5. Ovx^–^, ovariectomy; P4, progesterone.

Previous study demonstrated that gonococcal activated the NLRP3 inflammasome in THP-1 cells (Duncan et al., 2009; Li et al., 2019). The IL-1β (Figure 2D), IL-18 (Figure 2E) levels in the vaginal secretions in intact group were significantly higher than that observed in control group on the 5th day after inoculation. The Ovx^–^ group was slightly lower than that in the intact group. Meanwhile, the levels of IL-1β and IL-18 were obviously decreased in Ovx^–^ + P4 group (Figures 2D,E). To determine whether the IL-1β suppression was attributed to altered NLRP3 inflammasome activation, we assayed mRNA levels of NLRP3 (Figures 3A,B) in mice vaginal and cervical tissues. We found that mRNA levels of NLRP3 were increased in the intact group compared to that in the control group both in vaginal and cervical tissues. After treatment with P4, the mRNA levels of NLRP3 were decreased than that in the control group (Figures 3A,B). To further investigate the protein levels of NLRP3, relative fluorescence intensity of NLRP3 in vaginal and cervical tissues were obtained using ImageJ software. Results showed that NLRP3 expressions were repressed in P4-treated mice in cervical tissues. On the other hand, NLRP3 expressions changed mildly after P4 injected in vaginal tissues (Figures 4A,C). During the investigation of the mRNA levels of pro-IL-1β, the intact group was found to have higher levels than that in control group, and the significant decrease was seen after P4 injected both in the vaginal and cervical tissues (Figures 3C,D). Caspase-1 is a caspase-family member of intracellular cysteine proteases. Caspase-1 exists in cells as the inactive pro-caspase-1. After stimulation, pro-caspase-1 could cleave into 10 and 20 kDa subunits. To further investigate the caspase-1 activity, relative fluorescence intensity of caspase-1 p20 in vaginal and cervical tissues were obtained using Image J software, which showed that activated caspase-1 expressions were repressed in P4 injected mice both in vaginal and cervical tissues (Figures 4A,B). Traditionally, inflammasomes had mainly been studied in professional immune cells of the innate immune system, such as macrophages. To testify which cells were responsible for *N. gonorrhoea* infection *in vivo*, immunofluorescence of CD68 and IL-1β were detected in vaginal and cervical tissues, data showed that macrophage had partly involved in *N. gonorrhoea* infection *in vivo* (Figure 4D).

### P4 Inhibits *N. gonorrhoeae*-Induced NLRP3 Inflammasome Activation and IL-1β Secretion in THP-1 Macrophages

**FIGURE 5:**
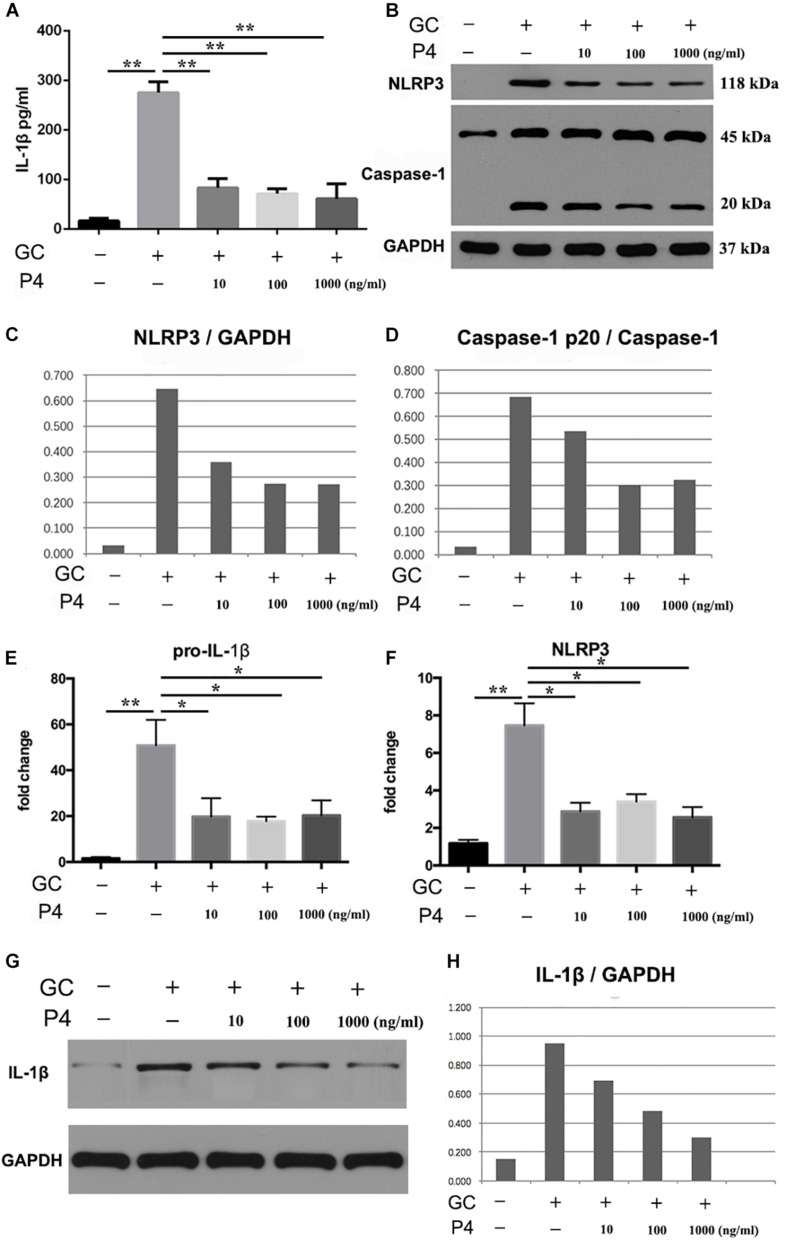
Progesterone inhibits *N. gonorrhoeae* -induced NLRP3 inflammasome activation and IL-1β secretion in THP-1 macrophages. **(A)** PMA-differentiated THP-1 cells were pre-treated with N. gonorrhoeae overnight and then stimulated with P4. Four hours later supernatants were analyzed by ELISA for IL-1β release. **(B)** Cells were selected to detect the protein levels of NLRP3 and Caspase-1p20. **(C,D)** Semi quantification analysis of NLRP3 and caspase-1p20 western blot films using ImageJ software. **(E,F)** The mRNA levels of pro-IL-1β and NLRP3 on N. gonorrhoeae infected human differentiated THP-1 macrophages. **(G,H)** Cell supernatants from activated THP-1 cells were evaluated for active IL-1β by Western blot, semi quantification analysis of active IL-1β western blot films using ImageJ software. *p < 0.05, **p < 0.01. Data are presented as the mean ± *SD*, *n* = 5. GC, *Neisseria gonorrhoeae*; P4, progesterone.

Next, we used human differentiated THP-1 macrophages to testify the effects of P4 on gonococci induced-inflammasome *in vitro*. To differentiate the THP-1 cells into macrophages, the cells were stimulated with Phorbol 12-myristate 13-acetate (PMA) for 72 h and then cultured for another 24 h before experiment. As previously described, in female serum P4 levels fluctuate between 3 and 30 nM. For a better simulation of the *in vivo* environment, we selected different P4 concentration (10, 100 ng/ml, 1 μg/ml) to stimulate the THP-1 macrophages. Figures 5A,G,H showed that the level of IL-1β was increased in gonococcal infected cells, and P4 decreased the IL-1β levels in a dose-independent way. Moreover, *N. gonorrhoeae* increased the synthesis of pro-IL-1β, and P4 decreased the level of pro-IL-1β in a dose-independent manner (Figure 5E). Data showed that P4 pretreatment significantly down-regulated gonococci-induced NLRP3 expression in both, the mRNA—(Figure 5F) and protein levels (Figures 5B,C) in a dose-independent manner. P4 also inhibited the activation of caspase-1 (Figure 5B) in a dose-independent manner (Figure 5D). Our results indicate that *N. gonorrhoeae* inhibits the activation of NLRP3 inflammasome by reducing the synthesis of pro-IL-1β, NLRP3, and the activity of caspase-1.

### P4 Inhibits *N. gonorrhoeae*-Induced Activation of NF-κB Signal Pathway, Resulting in Decreased Activation of NLRP3 Inflammasome and Attenuated Inflammation in THP-1 Cells

**FIGURE 6:**
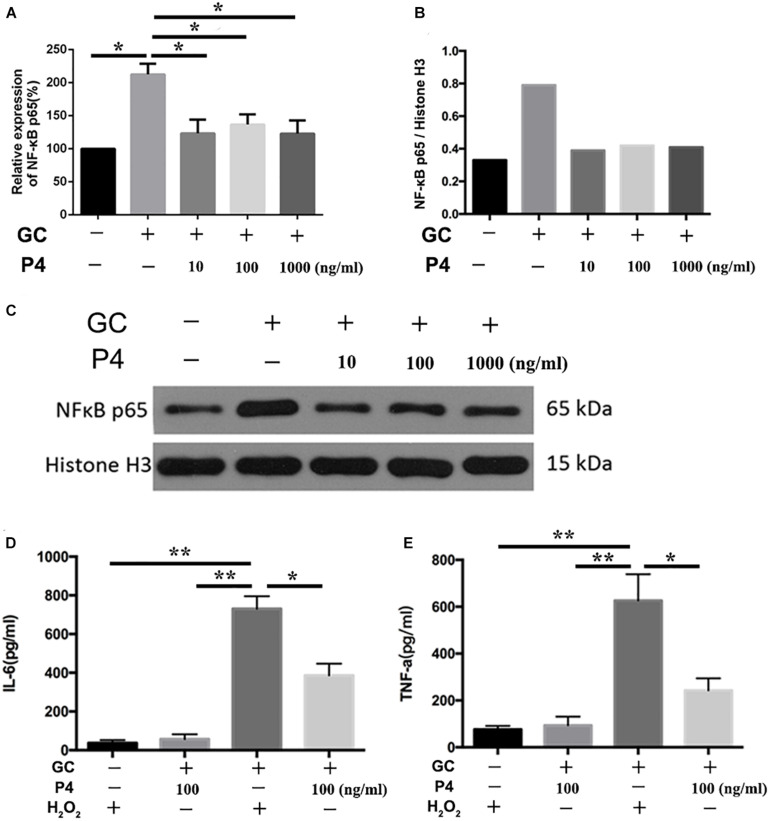
Progesterone inhibits the activation of NF-κB signal pathway. **(A)** PMA-differentiated THP-1 cells were pre-treated with *N. gonorrhoeae* overnight and then stimulated with P4. Four hours later, cells were selected to detect the mRNA levels of NF-κB p65. **(B)** Semi-quantification analysis of NF-κB western blot films using ImageJ software. **(C)** Phosphorylation of NF-κB and total NF-κB expression were detected by immunoblotting. Supernatants were analyzed by ELISA for IL-6 **(D)** and TNF-α release **(E)**. Data are representative or means ± *SD* of three independent experiments. *p < 0.05, **p < 0.01. GC, *Neisseria gonorrhoeae*; P4, progesterone.

NF-κB activation is the first signal required for the NLRP3 inflammasome activation (Swanson et al., 2019). Therefore, we studied whether P4 attenuates gonococci-induced NF-κB activation. Figure 6A shows that the mRNA levels of NF-κB p65 were increased in gonococcal-infected THP-1 macrophages, and P4 decreased the mRNA levels of NF-κB p65. P4 pretreatment was found to significantly attenuate gonococcal-stimulated phosphorylation of NF-κB detected by Western blot, while *N. gonorrhoeae* induced higher levels of NF-κB phosphorylation (Figures 6B,C). Since PMA was added to differentiate the THP-1 cells into macrophages, the basic levels of phosphorylation were relatively high. Figures 6D,E show that gonococci-induced IL-6 and TNF-α (other cytokines released by NF-κB) production were also significantly suppressed by P4.

### P4 Attenuates *N. gonorrhoeae*-Induced ROS Generation

**FIGURE 7:**
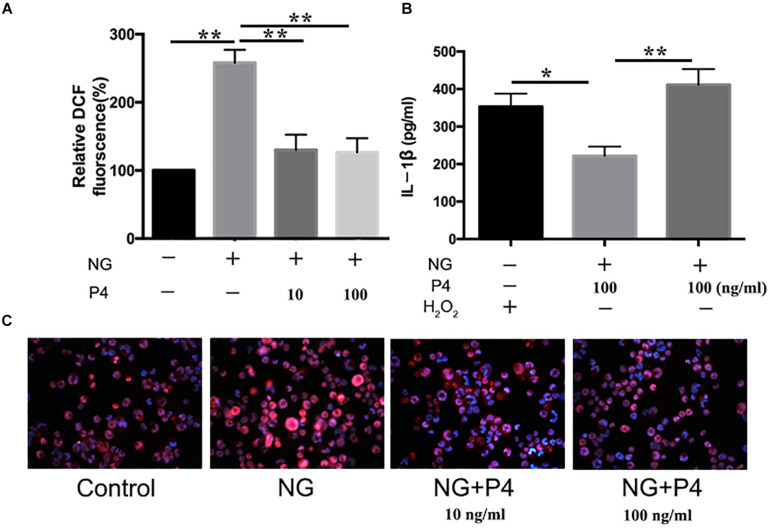
Progesteroneattenuates *N. gonorrhoeae*-induced ROS generation. **(A)** PMA-differentiated THP-1 cells were pre-treated with *N. gonorrhoeae* overnight and then stimulated with P4. Four hours later, oxidized DCF fluorescence was detected by fluorescence microscope, and semi-quantification analysis of the oxidized DCF fluorescence using ImageJ software. **(B)** P4 pre-treated differentiated THP-1 cells were stimulated with 5 mM H_2_O_2_ and *N. gonorrhoeae*, supernatants were analyzed by ELISA for IL-1β release. **(C)** Fluorescent images were visualized using an OLYMPUS Fluorescence microscope. Data are representative or means ± *SD* of at least three independent experiments. *p < 0.05, **p < 0.01. NG, Neisseria Gonorrhoeae; P4, progesterone.

ROS production, potassium outflow, calcium inflow, or lysosomal disruption are the second signal required by NLRP3 inflammasome activation (Franchi et al., 2012). While investigation if P4 attenuates the second signal for NLRP3 activation, we detected the ROS production reduced by gonococci infected P4-pretreated THP-1 macrophages. We found that ROS production was increased in gonococci-infected THP-1 macrophages (Figure 7A), and P4 pretreatment down-regulated gonococci-induced intracellular ROS production (Figures 7A,C). We found that H_2_O_2_ treatment efficiently reversed the inhibitory effect of P4 on the activation of IL-1β, IL-6, and TNF-α secretion induced by *N. gonorrhoeae* (Figures 6D,E, 7B). These results confirm that P4 reduced the gonococci-induced ROS production in THP-1 macrophages.

## Discussion

**FIGURE 8:**
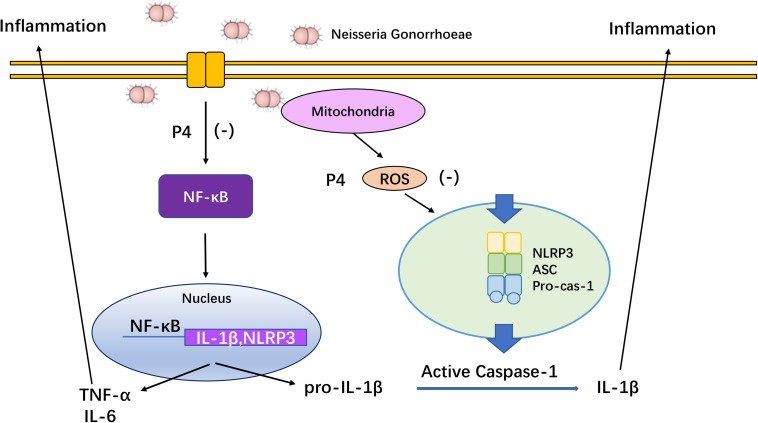
Overview of mechanisms by which progesterone inhibited NF-κB and NLRP3 inflammasome activated by *N. gonorrhoeae*. Progesterone inhibits *N. gonorrhoeae* induced-inflammation in murine models and macrophages through (1) inhibiting the activation of NF-κB signal pathway, thus decreasing NLRP3 and pro-IL-1β expression; (2) suppressing ROS production, thus inhibiting caspase-1 activation and IL-1β/IL-18 secretion.

Accumulating evidence suggests that the asymptomatic/subclinical gonococcal infections correlate with immunosuppressive signaling responses, particularly in innate PRRs and their downstream signals (Quillin and Seifert, 2018). In our previous studies, we found that the IL-1β and TNF-α levels in the cervical secretions of asymptomatic female patients with gonococcal infection were much lower than that of the symptomatic group while the average levels of serum P4 are negatively correlated with inflammation of cervical secretions (Wu et al., 2011). In the current study, P4 decreases cytokine release and the neutrophil infiltration in genital tissues of murine infection models. Furthermore, P4 down-regulates the mRNA and protein levels of NLRP3 and pro- IL-1β in vaginal and cervical tissues and represses caspase-1 activity. Moreover, P4 inhibits the NF-κB signal pathway and ROS production in THP-1 cells, which is consistent with the two signals required for activation of the NLRP3 inflammasome (Figure 8). Our results show that P4 suppresses the gonococci induced-inflammation, especially through the NLRP3 inflammasome pathway. These results have partly explained the mechanism of the pathogenesis of asymptomatic gonococcal infection in women.

P4 provides anti-inflammatory effects and decreases TLRs expression in epithelial cell lines from human fallopian tube (Zandieh et al., 2016). P4 also suppresses the release of IL-6, IL-8, TNF-α, and IL-10 in the amniotic epithelium from pregnant women induced by LPS (Flores et al., 2014). In murine macrophages, P4 inhibits the production of IL-6 and NO induced by LPS and CpG oligodeoxynucleotide (ODN), and up regulates TLR4 expression (Su et al., 2009). Several studies showed that P4 may increase the expression of TLR4, thus enhancing the innate immune response (Srivastava et al., 2007). Schatz et al. showed a significant increase in TLR4 expression in DCs during early pregnancy (Schatz et al., 2012). In the early stage of pregnancy, monocytes showed the highest TNF-α production induced by TLR4 (Ziegler et al., 2018). The reason that P4 has different modulatory effects on TLR4 expression is the fluctuation ofP4 levels during pregnancy, which is not identical to exogenous P4 supplement. Our results demonstrate that P4 decreases the inflammation in a dose-independent manner. However, other experiments have shown that P4 reduced nitric oxide synthase 2 (iNOS) in LPS- and TLR-4- induced macrophage in a dose-dependent manner (Menzies et al., 2011). The reason P4 attenuates the inflammation in a dose-independent manner is that there are different numbers of P4 receptors on THP-1 cells and macrophages.

P4 is considered to play stimulatory and suppressive roles in immune responses. Our study shows that P4 inhibits the activation of NF-κB in *N. gonorrhoeae* induced THP-1 cells. P4 can directly repress the transcription factor NF-κB and reduce inflammation by suppressing gene transcription downstream of the NF-κB pathway (Su et al., 2009; Lei et al., 2012). P4 inhibits IL-1β and cyclooxygenase-2 expression and the MAPK pathway through glucocorticoid receptors in human primary myometrial cells (Lei et al., 2015). In addition, the intracellular PRs expression in endometrial stem cells was reduced due to TNF-α or IL-1β stimulation (Grandi et al., 2016). However, not all studies proved that progesterone was an anti-inflammatory hormone. Research studies showed that inflammatory factors such as MIP-1, IL-6, and IL-8 were increased in cervical secretions in women taking DMPA (Deese et al., 2015; Fichorova et al., 2015).

Activation of the NLRP3 inflammasome requires two signals: stimulus activates NF-κB pathway to express pro-IL-1β and optimal NLRP3, and a second signal to assemble the NLRP3 inflammasome(Hayward et al., 2018). Inflammatory stimuli (such as TLRs activation) trigger the initiation of NLRP3 inflammasome by activating NF-κB, thereby inducing IL-1β and NLRP3 mRNA expression (Dinarello, 2009). Although previous studies have shown that lipooligosaccharide (LOS) can induce the pro-inflammatory cytokines through the TLR4-NF-κB pathway (Packiam et al., 2014; Casson et al., 2015), a recent study indicated that the first signal of the gonococciinduced-NLRP3 inflammasome activation was not dependent on TLR4, or TLR2 (Li et al., 2019). Strain MS11, an Opa positive strain, was showed to increase the IL-1β secretion in neutrophil (Sintsova et al., 2014). In our study, we show that gonococci activate the NF-κB, which is consistent with Li L-H‘s research (Li et al., 2019). Previous studies have shown that LOS variously induced pro-inflammatory cytokine expression in monocytes through MyD88, TRIF-dependent NF-κB and IRF-3 signaling (Figueiredo et al., 2009), and simultaneously activates NF-κB pathway in epithelial cells (Naumann et al., 1997; Muenzner et al., 2001; Dietrich et al., 2011). Besides, gonococci activates ERK1/2, JNK1/2, and p38 pathway in T84 human colonic epithelial-like cells (Howie et al., 2005, 2008) and activates ERK1/2 and JNK1/2 pathway in macrophages (Li et al., 2019).

Mitochondrial ROS, as a second signal, plays an important role in NLRP3 activation. Our study showed that P4 inhibits gonococci induced-mitochondrial ROS, this phenomenon may explain the negative regulation of NLRP3 inflammasome that was observed in our study. Previous studies showed that P4 could suppress the activation of macrophages and DCs (Jones et al., 2010) and inhibit the human neutrophil degranulation and free radicals’ generation (Nadkarni et al., 2016).

Our study confirmed that *N. gonorrhoeae* (strain MS11) up-regulated the mRNA levels of IL-1β, and secretion of IL-1β in THP-1 cells, which is consistent with some previous studies (Zhou et al., 2013; García et al., 2016; Li et al., 2019). However, findings reported by Killen García which showed lower secretions of IL-1β in N. gonorrhoeae (P9-17 strain)-infected human MDM (García et al., 2016). Previous study showed that P9-17 strain needs exogenous ATP to induce IL-1β secretion (García et al., 2016). Our and other studies showed that strain MS11 (this study), ATCC 49226 (Li et al., 2019), 1291 (Zhou et al., 2013), and FA1090 (García et al., 2016) induced IL-1β secretion without additional ATP treatment. This distinction can be explained by the significant difference between monocytes (Tsuchiya et al., 1980) and macrophages. In addition, the structural modifications of lipooligosaccharides (LOS) in different strains of *N. gonorrhoeae* leads to different inflammatory responses. For example, LOS from wildtype strain 1,291 induced IL-1β secretion in human THP-1 cells, while LOS from msbB-deficient strain 1,291 NG cannot. Also, modification of the LOS structures between these two strains were different.

Activation of Inflammasomes including NLRP1, NLRP3, NLRC4 is induced by various stimuli. Specific pathogen may stimulate corresponding inflammasome. Previous studies demonstrated that intracytoplasmic flagellin from salmonella was capable of inducing NLRC4-mediated CASP1 activation (Franchi et al., 2006; Miao et al., 2006) and homologs of T3SS Needle protein from pathogens including Shigella flexneri (MxiH) and enterohemorrhagic *E. coli* (EprI) (Yang et al., 2013) were also found to activate NAIP1–NLRC4. Meanwhile, other investigation proved that NLRP1 could be activated by Bacillus anthracis lethal toxin and Toxoplasma gondii (Boyden and Dietrich, 2006; Cavaillès et al., 2006; Newman et al., 2010). Although so far, the surface antigens of *Neisseria gonorrhoeae* including Opa, lipo-oligosaccharides have not been shown to activate NLRP1 and NLRC4 inflammasomes. Previous studies have reported that lipo-oligosaccharides from *Neisseria gonorrhoeae* could induce pyroptosis in mouse bone marrow macrophages (Duncan et al., 2009). From this perspective, is the NLRC4 inflammasome pathway involved in the pyroptosis caused by *Neisseria gonorrhoeae*? Whether NLRP1 and NLRC4 can be induced by *Neisseria gonorrhea* is an important question in our future investigation. Effect of progesterone on NLRC4 have been tested in cerebral ischemia. Studies demonstrated that progesterone reduced hypoxia-induced expression of NLRC4 and alleviated the damage of ischemic brain (Lammerding et al., 2016). Also, reduction of NLRC4 could be observed in primary cortical astrocytes and microglial cells after OGD with treatment of progesterone (Habib et al., 2020). The post-ischemic elevation of NLRC4 and its down-regulation by progesterone may shed light on the anti-inflammatory effects of gonadal hormones. There were no investigations concerning effect of progesterone on NLRP1, providing a chance of further study for us.

Collectively, our findings demonstrated that P4 down-regulates the expression of NLRP3 and pro-IL-1β, repressed the caspase-1 activation and IL-1β maturation. Moreover, P4 inhibits the NF-κB signal pathway and ROS production which is consistent with the two signals required by activation of the NLRP3 inflammasome. Our results have partly explained the mechanism of the pathogenesis of asymptomatic gonococcal infection in women, and may also provide new prevention strategies for asymptomatic/subclinical gonococcal infections and related diseases. For further understanding of P4 effect, a prospective area of research is on the specific and accurate progesterone receptors and downstream factors, which prevent the assembling of NLRP3 inflammasome.

## Data Availability Statement

The raw data supporting the conclusions of this article will be made available by the authors, without undue reservation, to any qualified researcher.

## Ethics Statement

The animal study was reviewed and approved by the USUHS Institutional Animal Care and Use Committee.

## Author Contributions

SZ, YZ, and LG performed experiment, analyzed the data, and wrote the manuscript. FW, BC, AA, XL, XD, JJ, XW, and MH performed experiment, analyzed the data, and prepared the images. HC, TC, and HC designed, conducted the research, interpreted data, and wrote the manuscript. All authors contributed to the article and approved the submitted version.

## Conflict of Interest

The authors declare that the research was conducted in the absence of any commercial or financial relationships that could be construed as a potential conflict of interest.
